# Mobile Phone Craving in Spain: Associations with Impulsivity, Anxiety, Gaming Problem, and Gambling Severity

**DOI:** 10.3390/bs16020234

**Published:** 2026-02-06

**Authors:** Jose de-Sola, Joan I. Mestre-Pintó, Víctor José Villanueva-Blasco, Hernán Talledo, Antonia Serrano, Gabriel Rubio, Fernando Rodríguez de Fonseca

**Affiliations:** 1 Grupo de Neuropsicofarmacología, Instituto de Investigación Biomédica de Málaga y Plataforma en Nanomedicina-IBIMA Plataforma BIONAND, Unidades Clínicas de Neurología y Psiquiatría, 29590 Málaga, Spain; jose.desola@desaludpsicologos.es (J.d.-S.); antonia.serrano@ibima.eu (A.S.); 2 De Salud Psicólogos, Centro de Psicología y Psicoterapia, Dr. Castelo 42, 28009 Madrid, Spain; 3 Addiction Research Group (GRAd), Neuroscience Research Program, Hospital del Mar Research Institute, 08003 Barcelona, Spain; jmestre@researchmar.net; 4 Faculty of Health Sciences, Valencian International University, Pintor Sorolla 21, 46002 Valencia, Spain; vjvillanueva@universidadviu.com; 5 Department of Marketing and Communication, Universidad Peruana de Ciencias Aplicadas, Lima 150140, Peru; pccmotal@ucp.edu.pe; 6 Departamento de Psiquiatria, Universidad Complutense de Madrid, Instituto de Investigación i+12, Hospital Universitario 12 de Octubre de Madrid, 28041 Madrid, Spain; grubiovalladolid@gmail.com

**Keywords:** craving, mobile phone, problematic use, dependence, impulsivity, anxiety, videogaming, gambling, prevalence, Mobile Phone Addiction Craving Scale (MPACS)

## Abstract

Craving for mobile phone use is increasingly discussed as a relevant feature of problematic engagement with digital technologies. This population-based study of 1601 Spanish adults examined psychological factors (impulsivity traits and affective symptoms) and behavioral correlates linked to mobile phone craving. Primary outcome: Mobile phone craving scale (MPACS). Secondary analyses: Associations between craving and impulsivity, anxiety, depression, Internet Gaming Disorder (IGD), gambling severity, and alcohol use. Craving measured with the MPACS was most common among younger participants (16–35 years old) and strongly related to greater daily phone use, heightened impulsivity, especially urgency and sensation seeking, and higher levels of anxiety and depressive symptoms. Among individuals who use their phones for gaming or gambling (n = 463), craving was strongly associated with IGD and gambling severity, suggesting that mobile phones may amplify involvement in these behaviors. Exploratory factor analyses consistently revealed four underlying dimensions—Reactive Impulsivity, Cognitive Impulsivity, Negative Emotions, and Addictive Behaviors—each contributing differently depending on craving intensity. Logistic regression analyses showed that anxiety, impulsivity, phone-use duration, and IGD scores independently predicted high craving levels. Overall, the findings highlight mobile phone craving as a clinically meaningful, multidimensional construct tied to emotional dysregulation and behavioral addiction. Assessing craving may help identify individuals at heightened risk for problematic technology use and related psychological difficulties.

## 1. Introduction

Problematic use of mobile phones and digital technologies has become a major concern in recent years. Smartphone overuse is increasingly conceptualized as a multidimensional phenomenon associated with impaired self-regulation, sleep disturbances, anxiety, and depressive symptoms (de-Sola et al., 2017b; Jahrami et al., 2022; W. Li et al., 2025; Lin et al., 2021; H. Tian & Wang, 2023; Y. Tian et al., 2025; Zhao et al., 2025). Beyond excessive frequency or duration of use, problematic mobile phone use reflects difficulties in controlling engagement with the device despite awareness of negative consequences. Growing evidence suggests that this pattern of use shares psychological and motivational mechanisms with other behavioral and substance-related addictions, including the presence of craving for mobile phone use (de-Sola et al., 2017a; Fernandez et al., 2020; Fransson et al., 2018). In this context, craving has emerged as a central construct for understanding persistent and dysregulated smartphone engagement.

Craving is considered a core motivational process across addictive disorders, reflecting heightened cue reactivity and an imbalance between reward-driven impulses and cognitive control. It can be defined as an unstoppable and uncontrollable desire that drives the use of a substance or behavior despite its negative and detrimental consequences. This impulsive loss of control may reflect the pursuit of gratification or positive reinforcement; it can also evolve into a compulsive pattern aimed at avoiding distress or dysphoria, consistent with negative reinforcement processes that sustain maladaptive use (Koob & Volkow, 2010/2010). Contemporary theoretical models emphasize that craving may be maintained through both positive reinforcement (seeking rewarding stimulation) and negative reinforcement (using the behavior to alleviate negative affect), a distinction that is particularly relevant in the context of smartphones, which provide constant access to rewarding and emotionally regulating digital cues (Robinson & Berridge, 2025). As such, craving represents a key mechanism linking emotional states, impulsive responding, and persistent technology use.

A growing body of literature indicates that problematic mobile phone use frequently co-occurs with a range of emotional and maladaptive behaviors. Meta-analytic evidence consistently links problematic smartphone use to elevated anxiety and depressive symptoms, supporting the view that negative affect and emotion dysregulation are central correlates of maladaptive engagement with mobile devices (Sohn et al., 2019). In parallel, behavioral addictions facilitated through mobile devices—such as online gaming, gambling, and compulsive checking behaviors—have expanded rapidly due to the accessibility and rewarding nature of digital environments (Nogueira-López et al., 2023; James et al., 2017, 2019; Lena-Acebo & García-Ruiz, 2019; Menéndez-García et al., 2022). Research across adolescent and adult populations indicates that problematic mobile phone use and internet-based gambling or gaming behaviors are highly prevalent and frequently coexist with emotional distress, impulsive responding, and maladaptive patterns of technology engagement (de-Sola et al., 2017b; James et al., 2017; Y. Li et al., 2020; Zhao et al., 2025).

Emotional dysregulation, reduced self-control, and maladaptive coping strategies have been identified as key mechanisms linking smartphone dependency with anxiety and depressive symptoms (de-Sola et al., 2017b; Zhao et al., 2025). Longitudinal research further suggests bidirectional relationships between problematic smartphone use and anxiety, indicating that emotional difficulties may both result from and exacerbate excessive use over time (Lin et al., 2021; Lu et al., 2025; Moreno et al., 2022). Among adolescents and young adults, additional vulnerabilities—such as social anxiety, alexithymia, and attentional difficulties—appear to amplify the risk of developing problematic patterns of engagement with smartphones, gaming, or social media platforms (Guo et al., 2025; S. Li et al., 2024; Menéndez-García et al., 2022). These findings align with broader behavioral addiction frameworks, in which impaired control, craving, and compulsive engagement represent central components sustaining continued use despite adverse outcomes (de-Sola et al., 2017a, 2017b).

From this theoretical framework, impulsivity traits—particularly urgency, defined as the tendency to act rashly under intense positive or negative affect—emerge as especially relevant correlates of mobile phone craving. Urgency may facilitate cue-driven, affect-dependent responding, while anxious or dysphoric states may increase reliance on smartphone use as a compensatory coping strategy, consistent with negative reinforcement pathways (Kardefelt-Winther, 2014; Rozgonjuk et al., 2018). Empirical syntheses consistently link problematic smartphone use with anxiety and depression, and recent studies highlight the importance of emotion-related impulsivity in problematic or addictive patterns of smartphone engagement (Cho & Kim, 2025; Elhai et al., 2017; Liu et al., 2024; Sohn et al., 2019). Importantly, impulsivity is not a unitary construct: urgency facets and sensation seeking have been repeatedly associated with addictive behaviors and problematic technology use, suggesting that affect-driven impulsivity may intensify craving responses, particularly when access to the device is restricted or delayed (Cho & Kim, 2025; Liu et al., 2024).

Mobile phones also serve as major access points for online gaming and gambling platforms, contributing to a growing overlap between problematic smartphone use, Internet Gaming Disorder (IGD), and gambling-related harm (James et al., 2019; Jo et al., 2020; Pallesen et al., 2021). Mobile formats facilitate rapid, portable access to rewarding activities, fostering frequent, intermittent engagement patterns and reinforcing habit loops that may strengthen craving and behavioral involvement. Studies in adolescents and clinical populations, including individuals diagnosed with ADHD, reveal heightened susceptibility to technology-related addictions, underscoring the interaction between impulsivity, reward sensitivity, and mobile digital environments (Demetrovics et al., 2022; El Archi et al., 2023).

Additional emerging research suggests that problematic smartphone use may co-occur with other health-risk behaviors, such as alcohol use. Although evidence remains limited, preliminary findings indicate that individuals exhibiting problematic or addictive technology use may show elevated impulsivity and vulnerability to substance use, potentially reflecting shared underlying mechanisms across addictive behaviors (de-Sola et al., 2019; Y. J. Kim et al., 2017; Na et al., 2017). As conceptualizations of behavioral addiction continue to evolve, the assessment of craving has gained increasing relevance for understanding excessive smartphone use. The development of specialized instruments such as the Mobile Phone Addiction Craving Scale (MPACS) has enabled more precise characterization of craving in the context of mobile technology (de-Sola et al., 2017a), facilitating investigation of the motivational and emotional processes that drive persistent engagement with smartphones and related digital behaviors.

The primary objective of this population-based study was to examine the associations between mobile phone craving, as measured by the Mobile Phone Addiction Craving Scale (MPACS), and a set of theoretically relevant correlates, including impulsivity traits, anxiety and depressive symptoms, phone-use intensity, and mobile-facilitated addictive behaviors such as gaming and gambling, in a large Spanish sample. Specifically, the study aimed to identify independent predictors of high levels of mobile phone craving using multivariable models and to assess whether craving covaried with the severity of mobile-facilitated behavioral addictions, namely Internet Gaming Disorder symptoms and gambling severity, among participants reporting engagement in these activities. In this context, the study further examined the associations between mobile phone craving, affective symptoms, impulsivity, alcohol use, and behavioral addictions within a single analytical framework, addressing the limited understanding of how these factors interact jointly in excessive smartphone engagement. As secondary objectives, the study sought to replicate and update key psychometric and measurement properties of the MPACS in a contemporary online population survey and to explore higher-order components integrating impulsivity traits, affective symptoms, and addictive behaviors. By clarifying the role of craving and emotional dysregulation within a broader spectrum of addictive behaviors, this work aims to improve understanding of the multidimensional mechanisms underlying problematic technology use, with potential implications for prevention and early detection, particularly among adolescents and young adults who may be especially vulnerable to maladaptive patterns of digital engagement.

## 2. Materials and Methods

### 2.1. Procedures and Instruments

The research was carried out via an online survey designed by our research group (de-Sola et al., 2017a, 2017b, 2019, 2026) and conducted by a sociological research company (ODEC S.L.) between May and June of 2025. The questionnaire, before being applied, was adjusted and finalized after a pilot study with several volunteer participants; these data were excluded from the final sample. These adjustments were related to the wording of some items, the response format, and the revision of some terms. The participants were sent an email invitation with a link to access the interview, which could be completed with interruptions, in as many sessions as necessary. Once the questionnaire had been completed, the link became unusable, making it impossible to reuse. The extraction and selection of the sample was performed using a database, with a total of 472,922 active collaborators in Spain, owned by the sociological research companies. The use of this type of database is common and frequent in sociological and market studies and is composed of individuals who, voluntarily or in exchange for a reward, participate in this type of research. In our case, the selection of our sample was performed following criteria that accounted for population balance and representativeness in relation to age, gender, main job or occupation, education level, and population center type (rural or urban) as variables of analysis, as well as geographic distribution in 17 of the 19 Spanish autonomous communities. Furthermore, all participants had to have at least their own mobile phone, an aspect that was evaluated through a filter question that conditioned the continuation of the questionnaire. In addition, several scales associated with factors identified in 2014 as relevant contributors to problematic phone use were included. Primary analyses focused on MPACS craving scores across the full sample (N = 1601). Secondary analyses examined associations with IGD (IGDS9-SF) and PGSI scores in the gaming/gambling subgroup (n = 463).

Thus, the questionnaire contained the following instruments.

#### 2.1.1. Mobile Phone Addiction Craving Scale (MPACS)

The MPACS is an 8-item scale that assesses the intensity of craving for mobile phone use in situations where the phone is unavailable. Items are rated on a 10-point Likert scale (1–10), with total scores ranging from 8 to 80 (higher scores indicate greater craving). The Spanish version has demonstrated excellent internal consistency (α > 0.90) and a unidimensional factor structure in community samples (de-Sola et al., 2017a, 2026).

#### 2.1.2. UPPS Impulsive Behavior Scale (UPPS-P Short Form)

The short form of the UPPS-P Impulsive Behavior Scale consists of 20 items across five subscales: negative urgency, positive urgency, lack of premeditation, lack of perseverance, and sensation seeking. Items are rated on a 4-point Likert scale. The short Spanish version has shown good psychometric properties, including a robust five-factor structure and acceptable to good internal consistency (α = 0.70–0.85) (Cándido et al., 2012; Verdejo-García et al., 2010).

#### 2.1.3. The Internet Gaming Disorder Scale—Short Form (IGDS9-SF)

It is a 9-item measure based on DSM-5 criteria for Internet Gaming Disorder. Responses are provided on a 5-point Likert scale and summed for a total severity score. The Spanish adaptation has demonstrated a unifactorial structure, excellent reliability (α > 0.90), and good construct validity in young adult samples (Beranuy et al., 2020; Sánchez-Iglesias et al., 2020).

#### 2.1.4. The Problem Gambling Severity Index (PGSI)

It is a 9-item scale assessing gambling-related problems over the past 12 months. Items are rated on a 4-point scale, yielding a total score indicative of gambling risk. The Spanish version has shown satisfactory construct validity, excellent internal consistency (α ≈ 0.97), and good convergent validity with DSM criteria (Lopez-Gonzalez et al., 2018).

#### 2.1.5. The Alcohol Use Disorders Identification Test—Consumption (AUDIT-C)

It is a 3-item screening tool assessing drinking frequency, quantity, and binge episodes. Scores range from 0 to 12, with higher scores indicating riskier drinking patterns. The Spanish AUDIT-C has demonstrated good screening properties for hazardous drinking (García Carretero et al., 2016).

#### 2.1.6. The State Form of the State—Trait Anxiety Inventory (STAI-S)

It is a 20-item scale measuring current anxiety symptoms. Items are rated on a 4-point scale (total score 20–80). The Spanish STAI-S (including short forms) is extensively validated with excellent reliability. (Buela-Casal & Guillén-Riquelme, 2017; Bush et al., 1998).

#### 2.1.7. The Beck Depression Inventory (BDI-21)

It is a 21-item self-report measure of depressive symptoms. Each item is scored 0–3 (total 0–63). The Spanish BDI-21 has established good reliability and validity for screening depressive symptoms (Bonicatto et al., 1998; Sanz et al., 2003).

A total of 1601 participants were included in the study, of whom 463 declared to be engaged in gaming/gambling through the mobile phone.

### 2.2. Ethics Statement

The study and recruitment protocols were approved by the Ethics Committee of the Hospital Regional Universitario de Málaga (Code FRF1, last approval: 1 September 2022) and were conducted in accordance with the Declaration of Helsinki (seventh revision, 2013, Fortaleza, Brazil). The internet-based survey included a specific informed consent form that provided a clear description of the nature and objectives of the research. Participants were required to sign the informed consent electronically to gain access to the questionnaire. No personal identifying information (e.g., name, address) was collected in order to preserve the anonymity of the respondents.

### 2.3. Sample and Participants

The sample consisted of 1601 participants (men and women aged 16 years and older) from across Spain. Age was analyzed both as a continuous variable and as predefined age bands matching the quota structure: 16–20, 21–25, 26–30, 31–35, 36–40, 41–45, 46–50, 51–55, 56–60, and 61–65 years. Throughout the manuscript, ‘younger participants’ refers to the 16–35-year range, consistent with prior work and the oversampling strategy used to ensure adequate precision in this high-risk segment. The sample distribution was proportional to the population size in 17 of the 19 Spanish autonomous communities, according to data from the National Institute of Statistics (Instituto Nacional de Estadística, INE, 2017). The two autonomous cities, Ceuta and Melilla, were excluded due to their small population size and limited representativeness. Table 1 presents the main characteristics of the participants. Most respondents lived in urban areas (over 50,000 inhabitants), while the remainder resided in rural areas or small population centers. Regarding age and gender, participant selection was determined by quotas to ensure equal representation of men and women. Given the sustained presence of problematic mobile phone users in the 16–35-year age group (de-Sola et al., 2017a, 2017b), the proportion of participants in this age range was increased with respect to previous studies. With respect to primary occupation, more than half of the participants were engaged in paid employment, while the rest were students, homemakers, unemployed individuals/retirees. In terms of educational level, over half had completed higher education, about 35% had secondary education, and approximately 8% had only basic education or no formal schooling.

### 2.4. Statistical Analysis

This research includes an analysis of the reliability of the instruments used, with a special focus on the review of the MPACS (Mobile Phone Addiction Craving Scale) for the assessment of craving with respect to previous studies (de-Sola et al., 2017a). Internal consistency was analyzed using Cronbach’s alpha, and internal validity was analyzed using factorial principal component analysis. For analysis of the characteristics of excessive craving, we selected the upper 25% based on MPACS scores obtained. This approach divided the population into low craving scores (mean score = 26.9, SD = 11.3, n = 1188) and high craving scores (mean score = 55.1, SD = 7.7, n = 413). Bivariate correlations were obtained between the scores of the different instruments for the initial assessment of associations, as well as in relation to the sociodemographic variables considered.

Significance of differences in qualitative and quantitative variables was determined by Fisher’s exact test (chi-square), Student’s *t* test, and Mann–Whitney U test, as required. Post hoc tests for multiple comparisons were performed using ANOVA followed by the Bonferroni correction test. Correlation analyses were performed using Spearman’s coefficient (rho). The normal distribution of the variables was assessed using the Lilliefors-corrected Kolmogorov–Smirnov test. Since most of the variables studied correlated, we performed principal component analysis with Promax rotation. Binary logistic regression analysis for analyzing factors predicting craving for the mobile phone was performed using Pearson’s chi-square (χ2) test with the Hosmer–Lemeshow test. Statistical analyses were carried out using GraphPad Prism version 5.04 and IBM SPSS Statistics version 22 (IBM, Armonk, NY, USA). A *p*-value < 0.05 was considered statistically significant.

## 3. Results

### 3.1. Reliability Studies of Instruments Used and Update of MPACS

Regarding the MPACS, we conducted a comprehensive psychometric reassessment of the MPACS to (a) replicate prior findings in a contemporary online-recruited sample and (b) evaluate potential changes in scale performance given the substantial evolution of smartphone features, usage patterns, and digital cue exposure over the past decade (2014–2025).

The scale consists of eight items with Likert-type responses, ranging from 1 to 10 points, depending on the conformity and degree of situational concern, with eight statements referring to a hypothetical present moment when a cell phone cannot be used. The overall score ranges from 8 to 80, with a mean of 34.22 (SD = 16.2), with higher scores indicating higher cravings. The MPACS also presented, from the beginning, good internal consistency (α = 0.916) as well as factorial unidimensionality, i.e., a single factor that explained 62.9% of the variance, which in this case indicated that craving was a unique construct. All items maintained factorial loads greater than 0.5, with the Kaiser–Meyer–Olkin measure of sampling adequacy being 0.93 (KMO = 0.926), while Bartlett’s test of sphericity provided a chi-square of 7500.21 (gl = 28; *p* < 0.0001), which confirmed the sampling adequacy as well as the suitability of the analysis. These results are very similar to those obtained in 2014 (de-Sola et al., 2017a).

Regarding the instruments used in this research, in general terms, they showed adequate internal consistency coefficients evaluated through Cronbach’s alpha (See Table 2), in line with those obtained in other studies with the same methodology (de-Sola et al., 2017a, 2017b, 2019, 2026).

### 3.2. Distribution of Scores by Age and Gender

Mean scores of each instrument distributed by age interval and gender can be examined in Table 3. MPACS scores are similar in both genders. As described previously, craving scores are greater in younger ages, decreasing thereafter (Age interval effect, *F*(9,1581) = 11.4, *p* < 0.001). PGSI scores were also higher in younger ages (*F*(9,444) = 4.30, *p* < 0.005) and were similar in both genders in all time intervals. This finding is mirrored when considering Internet Gaming Disorder evaluated with IGSD9-SF (*F*(9,444) = 3.81, *p* < 0.005). These results indicate that problematic mobile phone use, Internet Gaming Disorder, and problematic gambling are associated in young people (<35 years old). Regarding mood status, anxiety scores was more frequent in women (*F*(1,1581) = 5.96, *p* < 0.02) and also decreased with age (*F*(9,1581) = 5.66, *p* < 0.001); depression evaluated by BDI, displays the same pattern, with higher scores in women (*F*(1,1581) = 7.81, *p* < 0.001) and also had a temporal pattern with higher scores in young and aged people (*F*(9,1581) = 9.12, *p* < 0.001). Concerning impulsivity, scores were also affected by age (*F*(9,1581) = 19.01, *p* < 0.001) and gender (*F*(1,1581) = 4.96, *p* < 0.03). Alcohol consumption was higher in males (*F*(1,1581) = 27.61, *p* < 0.001) and was not affected by age.

### 3.3. Interrelationships Between Mobile Phone Craving, Pathological Gambling, Video Game Use Disorder, Impulsivity, and Mood States

In previous studies (de-Sola et al., 2017a, 2017b), we identified several variables that contribute to craving for mobile phone use, including age, hours of use, impulsivity, alcohol use, depression scores, and state anxiety. In the present study, we extended this work by examining the role of Internet Gaming Disorder and/or problem gaming. Table 4 presents the bivariate correlations among the scores obtained from the instruments described above, as well as age and mobile phone use hours. As reported previously, mobile phone craving is negatively correlated with age, with higher scores found in younger adults. In contrast, it is positively correlated with hours of use, impulsivity scores—particularly the positive and negative urgency dimensions—and state anxiety. As a novelty of this study, and among participants who reported using their mobile phone for gaming and/or gambling, we observed very strong correlations of craving with IGDS9-SF and PGSI scores, respectively. Correlation analyses using IGDS9-SF scores revealed a similar pattern, showing a strong association with PGSI scores and a positive correlation with the sensation-seeking dimension of the UPPS impulsivity scale. Interestingly, both IGDS9-SF and PGSI scores displayed clear correlations with the AUDIT-C alcohol use disorder screening score.

Based on this information, we performed a principal components analysis with Promax rotation, considering only the population declaring to be engaged in gambling and/or gaming. Table 5 shows the pattern matrix obtained for the general set of participants, while Table 6 shows the same pattern matrix in the upper 25% of craving scores.

When considering the whole sample of gambling/gaming participants (n = 463, Table 5), the factorial analysis fit was good. All items maintained factorial loads greater than 0.4, with the Kaiser–Meyer–Olkin measure of sampling adequacy being 0.78 (KMO = 0.784), while Bartlett’s test of sphericity provided a chi-square of 2246.4 (gl = 78; *p* < 0.001), which confirmed the suitability of the analysis. Four components explained 64.1% of the variance. The first one, explaining 32.4% of the variance, grouped being young and displaying high scores for impulsivity on the dimensions of negative urgence, positive urgence, and sensation seeking, so we termed it “Reactive Impulsivity”. The second component, explaining 13.9% of variance, grouped being women and having greater scores for depression and anxiety, so we termed it “Negative Emotions”. The third one grouped the UPPS dimensions of Lack of Perseverance and Lack of Premeditation. It explained 9.7% of the variance, and we named it “Cognitive Impulsivity”. Finally, the fourth component grouped being a man and having higher scores in gambling (PGSI), gaming (IGDS9-SF), and alcohol consumption (AUDIT_C). We named it “Addictive Behaviors,” and it accounts for 8% of the variance.

Considering the subpopulation with higher craving scores (upper 25 percent scores of MPACS, Table 6), the factorial analysis fit was also good. All items maintained factorial loads greater than 0.4, with the Kaiser–Meyer–Olkin measure of sampling adequacy being 0.75 (KMO = 0.754), while Bartlett’s test of sphericity provided a chi-square of 744.9 (gl = 78; *p* < 0.001), which confirmed the suitability of the analysis. Again, the same four components explained 62.9% of the variance, although their contribution differs from that of the whole population, priming the addictive component over other contributing factors. The first one explained 30.6% of the variance, and it grouped being male and having higher scores in gambling (PGSI), gaming (IGDS9-SF), and alcohol consumption (AUDIT_C), with an interesting contribution of higher depression scores. We named it “Addictive Behaviors”. The second one accounted for 15.1% of the variance and grouped being young and having higher scores for the UPPS dimensions of Lack of Perseverance and Lack of Premeditation. We named it “Cognitive Impulsivity”.

The third component grouped being young, with a high number of hours of use of the mobile phone and displaying high scores for impulsivity on the dimensions of positive urgence and sensation seeking, so we termed it “Reactive Impulsivity”. It contributed with a 9.5% to the variance. Finally, the fourth component, explaining 7.7% of variance, grouped being women and having greater scores for depression and anxiety, so we termed it “Negative Emotions”.

Based on these results, we performed binary logistic regression in the participants with higher craving scores (upper 25%), considering a) the whole population of the study without including gambling/gaming scores, and b) the participants recognizing the use of the mobile phone for gaming and/or gambling. Table 7 shows the fit of both models.

**Figure 1:**
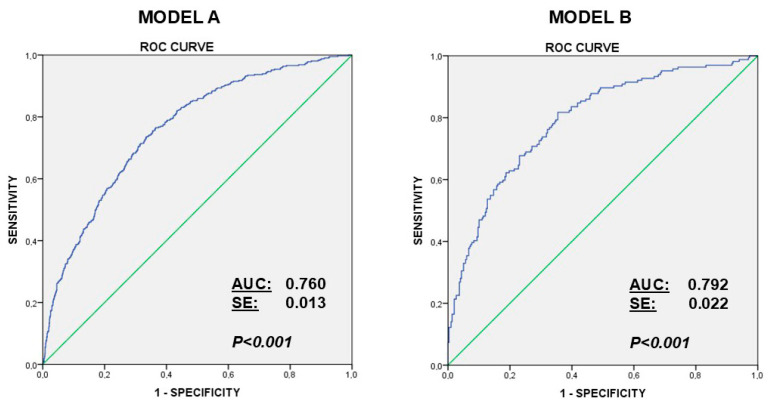
ROC curves for multivariate predictive models of craving mobile phone use (category upper 25% craving scores). **Model A**, performed with data of all participants, includes, in the final analysis, the variables, the number of hours of use of the mobile phone, STAI-S anxiety score, four UPPS dimensions of impulsivity (negative urgence, positive urgence, lack of premeditation, and sensation seeking), and AUDIT-C scores for alcohol consumption. **Model B**, performed with the scores for gambling (PGSI) and gaming (IGDS9-SF), includes the number of hours of use of the mobile phone, STAI-S anxiety score, BDI depression scores, positive urgency dimension of impulsivity, and Internet Gaming Disorder score (IGDS9-SF).

In model A (the upper 25% of craving score), the variables included in the first step were “age”, “ Gender”, “Hours of Use”, “Educational Level”, “Anxiety Score (STAI)”, “Depression Score (BDI)”, “UPPS-Negative Urgence”, “UPPS-Positive Urgence”, “UPPS-Lack of Perseverance”, “UPPS-Lack of Premeditation”, “UPPS-Sensation Seeking”, and “Alcohol Consumption (AUDIT-C score)”. In the model B (upper 25% craving scores declaring gaming/gambling), the variables included in the first step were “age”, “ Gender”, “Hours of Use”, “Educational Level”, “Anxiety Score (STAI)”, “Depression Score (BDI)”, “UPPS-Negative Urgence”, “UPPS-Positive Urgence”, “UPPS-Lack of Perseverance”, “UPPS-Lack of premeditation”, “UPPS-Sensation Seeking”, “Alcohol Consumption (AUDIT-C score)”, “Problem Gambling Severity Index” (PGSI score) and “Internet Gaming Disorder” (IGDS9-SF score.) The models were prepared using the backward stepwise method. The predictive covariates were (a) restricted to seven in the general model A (“Hours of Use”, “Anxiety Score (STAI)”, “UPPS-Negative Urgence”, “UPPS-Positive Urgence”, “UPPS-Lack of Perseverance”, “UPPS-Sensation Seeking” and “Alcohol Consumption (AUDIT-C score)”.), and to (b) five in Model B (Gambling/Gaming population: “Anxiety Score (STAI)”, “Depression Score (BDI)”, “UPPS-Positive Urgence”, “Hours of Use” and “Internet Gaming Disorder” (IGDS9-SF score). In Model A, The Hosmer–Lemeshow test indicated good calibration (X^2^ = 3.98; df = 8; *p* = 0.86). ROC curve analysis (AUC = 0.760) indicated an acceptable discrimination power (Figure 1). In Model B, The Hosmer–Lemeshow test indicated good calibration (X^2^ = 6.31; df = 8; *p* = 0.612). ROC curve analysis (AUC = 0.792) indicated a high discrimination power (Figure 1).

## 4. Discussion

This population-based study examined psychological factors (impulsivity traits and affective symptoms) and behavioral correlates linked to mobile phone craving in Spain, integrating measures of impulsivity, affective symptomatology, and behavioral addictions within a large and representative sample. The findings support the conceptualization of mobile phone craving as a clinically relevant construct (de-Sola et al., 2017b; Gao et al., 2024; Shi et al., 2025), analogous to craving phenomena described in substance use and behavioral addictions and highlight a constellation of interacting vulnerability factors—including impulsive personality traits, negative affect, and co-occurring addictive behaviors—that converge to elevate mobile phone craving intensity and problematic use patterns (Koob & Volkow, 2010/2010; Miele et al., 2023). The convergence of these domains is reflected in the correlation matrices and factor analyses (Table 4, Table 5 and Table 6, Figure 1) that—taken together—provide a strong empirical basis for considering mobile phone craving as a multidimensional indicator of addiction vulnerability. Notwithstanding the limitations described at the end of the discussion, a major strength of the study lies in its large sample size and the representativeness of the Spanish population, with coverage spanning virtually the entire national territory, providing robust population-level evidence on mobile phone craving and its psychological correlates.

### 4.1. Considering Current Addiction Models, Craving for the Mobile Phone Emerges as a Solid Construct

Craving is widely regarded as a hallmark feature of addiction, central to diagnostic criteria across substance use disorders and behavioral addictions (e.g., gambling disorder) and strongly linked to relapse and compulsive engagement (Gao et al., 2024; Koob & Le Moal, 1997; Mallorquí-Bagué et al., 2023; Miele et al., 2023; Robinson & Berridge, 2025; Toulami et al., 2025). In the current study, craving, as operationalized through the Mobile Phone Addiction Craving Scale (MPACS, exhibited strong psychometric reliability and unidimensionality, replicating previous validations and suggesting that the constant evolving changes in the digital ecosystem are not changing the impact on the need to use the mobile device. Craving scores were highest in younger participants (Table 3). This distribution mirrors developmental patterns described in addiction neuroscience, in which heightened reward sensitivity and reduced prefrontal control during adolescence and young adulthood (Chick, 2015; Fryt et al., 2021) potentiate compulsive engagement, as reflected here by elevated UPPS dimensions of Lack of Premeditation and Lack of Perseverance. The findings reinforce that mobile phone craving behaves analogously to craving in substance and behavioral addictions, displaying strong associations with hours of use, affective dysregulation, and high-urgency impulsivity (Table 4). This supports theoretical frameworks in which craving reflects dysregulated motivational circuitry and heightened responsiveness to conditioned cues—here, the constant availability, social reinforcement, and reward unpredictability of the smartphone (Mallorquí-Bagué et al., 2023; Toulami et al., 2025). The strong positive correlations of craving with both Internet Gaming Disorder (IGD) and problem gambling severity (PGSI) in the gaming/gambling subgroup (Table 4) further suggest that craving for the device may partially reflect craving for the addictive activities facilitated by the device itself, aligning with prior studies emphasizing the enabling role of smartphones in behavioral addictions (James et al., 2017, 2019).

### 4.2. Impulsivity Dimensions: Cognitive and Reactive Components

The UPPS-P model has proven crucial for delineating the nuanced roles of impulsivity facets in addictive behaviors (Albein-Urios et al., 2014; Y. Kim et al., 2016). In this study, impulsivity consistently predicted craving intensity, with the strongest associations emerging for negative urgency, positive urgency, and sensation seeking—three dimensions linked to affect-driven rash action and reward pursuit. These dimensions loaded prominently in the “Reactive Impulsivity” component in the full gaming/gambling subgroup (Table 5) and in the “Reactive Impulsivity” and “Cognitive Impulsivity” factors in the high-craving group (Table 6), indicating that both reactive and cognitive dimensions contribute meaningfully to craving vulnerability. The logistic regression models further underscore the independent predictive power of impulsivity. In Model A (general population), negative urgency, positive urgency, lack of perseverance, sensation seeking, and hours of use were all significant predictors of high craving (Table 7). This pattern aligns with literature demonstrating that urgency traits—acting under strong emotions—consistently predict behavioral addictions, including problematic smartphone use, IGD, and gambling (Billieux et al., 2021; Eben et al., 2023; Pérez-de-Albéniz-Garrote et al., 2021). Cognitive impulsivity traits (lack of premeditation and perseverance) also emerged as relevant, particularly in the high-craving subgroup factor analysis (Table 6), which grouped these dimensions within a distinct “Cognitive Impulsivity” component. These findings highlight that craving may arise from a combination of rapid affect-driven urges and insufficient top-down cognitive regulation.

### 4.3. Negative Affect, Anxiety, and Depression as Drivers of Craving

A consistent pattern across analyses indicates that craving is closely linked to negative affective states, particularly anxiety and depression, as stated in the hedonic homeostasis dysregulation described for substance use disorders (Koob & Le Moal, 1997). Bivariate correlations (Table 4) show robust associations of craving with both STAI-S anxiety scores and BDI depressive symptoms, and both anxiety and depression contributed prominently to the “Negative Emotions” factor in Table 5 and Table 6. These findings support a negative reinforcement model in which mobile phone use serves as a maladaptive coping strategy aimed at alleviating distress (Cho & Kim, 2025), consistent with models of craving in substance use and gambling disorders (Mallorquí-Bagué et al., 2023). Remarkably, this negative affect impacts in a gender-dependent manner: females scored higher on both anxiety and depressive symptoms across most age groups (Table 3), consistent with epidemiological data showing a greater burden of internalizing disorders among women (van Loo et al., 2023). However, craving did not show a gender imbalance, suggesting that while negative affect contributes significantly, it is not the sole driver. In the regression analyses, anxiety emerged as a significant positive predictor of craving in both Model A and Model B (Table 7), underscoring its central role. Interestingly, depression appeared as a negative predictor in Model B, possibly reflecting statistical suppression effects once gaming/gambling scores were included—consistent with the high intercorrelation among IGD, PGSI, and mood symptoms. Overall, the data strongly support that craving is intertwined with emotional dysregulation. This aligns with broader evidence linking problematic smartphone use with anxiety, depressive symptoms, and maladaptive emotion regulation (Y. Li et al., 2020; Y. Tian et al., 2025).

### 4.4. Behavioral Addictions and Alcohol Use as Facilitators of Mobile Phone Craving

One of the most salient findings is the powerful role of behavioral addictions—particularly IGD and gambling—in amplifying craving. In the gaming/gambling subgroup, IGD9-SF and PGSI scores displayed very strong correlations with MPACS scores (Table 4), far stronger than associations observed with any other psychological variable. This suggests a shared addiction phenotype, in which craving for the phone reflects an underlying drive to engage in the addictive activity accessed through the phone (Fransson et al., 2018; James et al., 2017; Y. Li et al., 2023). Factor analyses corroborate this interpretation. In the high-craving group, the primary factor was *“Addictive Behaviors,”* comprising IGD, PGSI, and alcohol consumption—a profile strongly associated with male gender (Table 6). This pattern suggests that in individuals with high craving, the phone functions as a delivery mechanism for multiple addictive behaviors, amplifying both frequency and intensity of engagement. The inclusion of IGD9-SF as a strong independent predictor of craving in regression Model B (Table 7) highlights the centrality of gaming-related reward mechanisms in the smartphone craving pathway. Alcohol use (AUDIT-C) also correlated significantly with craving in the entire sample (Table 4) and might serve as a predictor in Model A (Table 7), aligning with evidence showing shared neurobehavioral vulnerabilities between alcohol use disorders and behavioral addictions (de-Sola et al., 2017a; Y. J. Kim et al., 2017; Na et al., 2017). Together, these findings highlight the importance of considering comorbid addictive patterns when evaluating smartphone craving and problematic use, as the interplay of multiple addictive behaviors likely potentiates craving through shared impulsive and affective dysregulation mechanisms.

### 4.5. Academic and Clinical Implications

From a clinical perspective, assessing mobile phone craving may help identify individuals at elevated risk for problematic engagement with mobile-facilitated addictive behaviors. Brief screening of craving, together with impulsivity (urgency) and anxiety symptoms, could support stepped-care decisions, such as psychoeducation on digital habits, CBT-informed strategies targeting emotion regulation and cue-reactivity, and interventions focusing on affect-driven impulsive responding. Meta-analytic evidence supports the close link between problematic smartphone use and anxiety/depression, underscoring the importance of integrating mental health assessment rather than treating excessive phone use as an isolated habit (Sohn et al., 2019).

Academically, our findings align with addiction models emphasizing that craving reflects dysregulated motivational states shaped by reinforcement learning and environmental cues. Smartphones uniquely concentrate cues and immediate access to rewarding activities, including gaming and gambling, which may strengthen habit loops and craving-driven engagement (Robinson & Berridge, 2025; James et al., 2019). Accordingly, prevention efforts may benefit from targeting younger groups, where problematic smartphone use tends to peak, while also considering device-specific pathways to gaming and gambling harms.

### 4.6. Limitations of This Study

Several limitations of this study should be acknowledged. First, the cross-sectional and population-based design precludes causal inference regarding the relationships between impulsivity, affective symptoms, and mobile phone craving, underscoring the need for longitudinal and experimental research to clarify directionality and potential mediating mechanisms. In addition, population-based surveys cannot provide sufficiently large samples of individuals with pathological gambling, limiting the ability to examine bidirectional associations between mobile phone craving and gambling severity; future studies should therefore include targeted recruitment of mobile phone users with gambling disorder. Second, all data were collected through an online self-report questionnaire, which, despite being an efficient and widely used methodology, is inherently subject to recall bias, social desirability effects, and the lack of objective behavioral assessments. This limitation is compounded by participants’ subjective awareness of their own problematic mobile phone use, which may introduce additional reporting bias. Third, the online panel quota design involved intentional oversampling of participants aged 16–35 years, which may restrict the generalizability of unweighted descriptive estimates to the broader population, a known limitation of nonprobability online panels even when quotas are applied. Finally, the latent-structure analyses conducted in this study are exploratory in nature and should be replicated and extended using confirmatory methods and invariance testing across key demographic groups.

## 5. Conclusions

The present study highlights the widespread and consolidated use of mobile phone technology in Spain, as well as the lack of control over such use when it is associated with impulsivity, anxiety, or behavioral addictions such as Internet Gaming Disorder and/or problem gambling. Taken together, the findings of this study demonstrate that mobile phone craving is not an isolated behavioral phenomenon but rather a complex addiction-related construct situated at the intersection of impulsivity, emotional dysregulation, and co-occurring addictive behaviors. The facilitating role of mobile phones in videogaming clearly emerges, whereas further research is needed to elucidate their contribution to problem gambling, particularly among young males. The influence of negative mood states is also noteworthy—especially among females—and additional studies are required to determine whether mobile phone technology is used to alleviate anxiety and mitigate depressive symptoms, or whether this relationship reflects an independent associated phenomenon. Overall, this study suggests the importance of considering mobile phone craving as a relevant problem that may serve as an indicator of other psychopathological conditions, helping to implement more effective interventions.

## Figures and Tables

**Table 1:** Sociodemographic data for the sample (2025).

Autonomous Communities		Population by Inhabitants		Age	
Andalusia	18.4%	Up to 2000	2.4%	16 to 20 years	13.6%
Aragon	2.9%	From 2001 to 5000.	3.5%	21 to 25 years	25.9%
Asturias	2.1%	From 5001 to 10000	4.9%	26 to 30 years	11.7%
Balearic Islands	1.9%	From 10,001 to 50,000	20.4%	31 to 35 years	18.4%
Canary Islands	4.1%	From 50,001 to 200,000	21.9%	36 to 40 years	2.4%
Cantabria	1.1%	From 200,001 to 500,000.	5.5%	41 to 45 years	3.4%
Castilla La Mancha	4.4%	More than 500,000	41.5%	46 to 50 years	5.4%
Castilla León	5.4%			51 to 55 years	9.2%
Catalonia	**16.6%**	**Population size**		56 to 60 years	4.7%
Extremadura	2.4%	Rural areas	31.1%	61 to 65 years	5.3%
Galicia	6.1%	Urban areas	68.9%		
La Rioja	0.4%			**Mean age in years**	34.2
Madrid	14.7%	**Gender**		Standard Deviation age in years	14.2
Murcia	2.8%	Women	50.1%		
Navarre	1.2%	Men	**49.9%**	**Main activity**	
Basque Country	4.8%			Working	59.8%
Valencian Community	10.7%	**Studies**		Studying	22.7%
		Higher, university	56.5%	Housework	3.2%
**Mean Hours of use of the mobile phone**	4.8	Middle, high school	35.1%	No activity, unemployed, retired	14.2%
Standard Deviation (hr)	3.2	Basic or uneducated	8.4%		

**Table 2:** Internal consistency of the instruments used.

Instrument	Mean	SD	Median	Maximum	Minimum	Cronbach’s Alpha	Number of Cases
**MPACS**	34.23	16.19	34	80	8	0.916	1601
**PGSI**	4.54	6.13	1	27	0	0.946	463
**IGDS9-SF**	15.38	8.38	10	45	9	0.959	463
**STAI-S**	41.49	11.33	41	80	20	0.931	1601
**BDI-21**	14.38	14.73	10	63	0	0.877	1601
**UPPS-SF (Total)**	41.61	8.37	42	70	21	0.827	1601
**UPPS—Negative Urgence**	8.06	2.73	8	16	4	0.773	1601
**UPPS—Positive Urgence**	8.10	2.45	8	16	4	0.662	1601
**UPPS—Lack of Premeditation**	9.02	2.65	9	16	4	0.783	1601
**UPPS—Lack of Perseverance**	8.74	2.67	9	16	4	0.789	1601
**UPPS—Sensation Seeking**	7.71	2.81	8	16	4	0.809	1601
**AUDIT-C**	5.09	2.10	5	15	3	0.733	1601

**Table 3:** Mean scores of the instruments used, stratified by age and gender.

	Age Interval									
	16–20	21–25	26–30	31–35	36–40	41–45	46–50	51–55	56–60	61–65
**MPACS**										
**Males**	40.8 (14.8)	37.9 (15.0)	39.5 (16.4)	33.5 (15.7)	34.8 (16.1)	30.6 (15.1)	31.1 (15.9)	28.5 (15.9)	24.1 (13.8)	24.7 (15.1)
**Females**	37.4 (14.6)	37.5 (16.7)	35.6 (16.0)	33.4 (15.3)	27.9 (15.7)	30.1 (13.6)	26.6 (17.7)	31.6 (16.6)	31.4 (18.1)	26.2 (17.8)
**PGSI**										
**Males**	8.3 (7.3)	6.7 (6.2)	2.3 (3.5)	4.2 (5.9)	5.4 (7.3)	2.3 (3.9)	2.1 (3.9)	2.0 (4.9)	1.4 (3.2)	0.5 (1.2)
**Females**	6.6 (6.3)	5.1 (6.2)	3.4 (5.4)	2.7 (5.2)	ND	6.0 (10.3)	3.0 (5.1)	1.4 (4.2)	3.7 (8.5)	0 (0)
**IGDS9**										
**Males**	20.1(9.6)	18.8 (8.7)	12.8 (5.2)	14.5 (7.1)	15.4 (8.1)	11.5 (5.4)	12.6 (7.4)	12.1 (6.8)	11.5 (5.9)	9.1 (0.5)
**Females**	18.7 (8.9)	16.3 (7.9)	12.8 (8.1)	14.4 (8.0)	ND	15.3 (10.1)	13.7 (8.1)	11.4 (5.1)	15.8 (13.5)	12.0 (6.0)
**STAI-S**										
**Males**	41.7 (10.7)	42.5 (11.3)	42.6 (11.3)	40.7 (10.9)	41.9 (12.7)	41.7 (9.2)	38.2 (10.9)	39.0 (12.4)	38.4 (10.8)	34.6 (9.2)
**Females**	45.3 (10.9) *	44.0 (11.5)	41.7 (11.2)	41.3 (10.9)	46.6 (8.2)	40.1 (14.9)	41.2 (13.1)	39.0 (11.3)	44.8 (11.8) *	36.8 (10.2)
**BDI-21**										
**Males**	15.8 (14.6)	16.9 (16.8)	14.2 (20.6)	11.8 (14.9)	16.1 (16.7)	8.9 (9.7)	9.5 (11.1)	9.1 (11.4)	8.7 (9.7)	7.6 (8.1)
**Females**	23.6 (18.9) *	17.2 (16.7)	14.3 (20.9)	14.7 (13.0)	20.9 (9.3)	8.8 (8.8)	10.6 (11.4)	11.5 (10.2)	15.4 (13.6) *	10.4 (7.1)
**UPPS**										
**Males**	45.3 (10.9)	44.9 (7.9)	42.5 (7.8)	40.6 (6.5)	42.0 (8.2)	40.0 (7.6)	37.5 (8.3)	39.3 (8.4)	38.5 (7.5)	38.0 (7.5)
**Females**	45.8 (10.8)	43.0 (8.1) *	41.4 (8.4)	42.0 (8.2) *	40.6 (6.5)	35.1 (8.0) *	39.0 (6.3)	36.3 (11.3) *	39.2 (10.1)	37.0 (6.5)
**AUDIT**										
**Males**	5.0 (2.3)	5.4 (2.5)	5.6 (2.2)	5.2 (2.0)	5.7 (2.3)	5.4 (1.9)	5.6 (2.2)	5.7 (2.1)	5.7 (1.9)	5.5 (1.8)
**Females**	4.8 (2.4)	5.0 (1.8) *	4.8 (2.0) *	4.6 (1.7) *	4.6 (1.6)	4.7 (2.5)	5.1 (1.8)	4.8 (2.0) *	4.2 (1.7) *	4.3 (1.6) *

Mean score value and standard deviation (in parentheses) of the instruments used. ND: No data. (*) *p* < 0.05. Females versus males of the same age interval.

**Table 4:** Correlations of MPACS, PGSI, and IGDSP with the main instruments of the study.

Variable	MPAC Score	PGSI Score	IGDSP Score
**Age**	−0.255 **	−0.352 **	−0.332 **
**Hours of Use**	0.394 **	0.296 **	0.320 **
**MPACS Score**		0.501 **	0.511 **
**PGSI Score**	0.501 **		0.815
**IGDSP Score**	0.511 **	0.815 **	
**STAI-S Score**	0.318 **	0.321 **	0.390 **
**BDI-21 Score**	0.255 **	0.349 **	0.434 **
**UPPS Score**	0.398 **	0.534 **	0.549 **
**UPPS-NU**	0.368 **	0.394 **	0.447 **
**UPPS-PU**	0.318 **	0.380 **	0.393 **
**UPPS-LPREM**	0.128 **	0.218 **	0.213 **
**UPPS-LPERS**	0.178 **	0.116 *	0.084
**UPPS-SS**	0.241 **	0.449 **	0.460 **
**AUDIT-C Score**	0.120 **	0.318 **	0.301 **

Spearman correlations between craving dimension, problematic gambling and internet gaming disorders, with the main instruments used in the study. Negative correlations are depicted in red, possitive correlations in green. (*) *p* < 0.05, (**) *p* < 0.01.

**Table 5:** Principal component analysis of gaming/gambling among participants.

Pattern Matrix for the Whole Sampling (n = 463)				
	Component			
	1 Reactive Impulsivity	2 Negative Emotions	3 Cognitive Impulsivity	4 Addictive Behavior
**Variance Explained**	32.4%	13.9%	9.7%	8.0%
**Age**	−0.758			
**Woman/Men**		−0.0542		0.637
**BDI Score**		0.796		
**STAI Score**		0.827		
**UPPS—Negative Urgence**	0.620			
**UPPS—Positive Urgence**	0.728			
**UPPS—Lack of Premeditation**			0.862	
**UPPS—Lack of Perseverance**			0.862	
**UPPS—Sensation Seeking**	0.852			
**IGSD9_SF Score**				0.544
**PGSI Score**				0.578
**AUDIT_C Score**				0.781

**Table 6:** Principal component analysis of gaming/gambling among participants in the upper 25% of craving scores.

Pattern Matrix (N = 127)				
	Component			
	1 Addictive Behavior	2 Cognitive Impulsivity	3 Reactive Impulsivity	4 Negative Emotions
**Variance Explained**	30.6%	15.1%	9.5%	7.7%
**Age**		−0.422	−0.884	
**Woman/Men**	0.585			−0.606
**Hours of Use**			0.406	
**BDI Score**	0.407			0.662
**STAI Score**				0.788
**UPPS—Positive Urgence**			0.404	
**UPPS—Lack of Premeditation**		0.913		
**UPPS—Lack of Perseverance**		0.860		
**UPPS—Sensation Seeking**			0.586	
**IGSD9_SF Score**	0.759			
**PGSI Score**	0.717			
**AUDIT_C Score**	0.731			

**Table 7:** Binary logistic regression of factors predicting the craving for the mobile phone.

Model	Variable	B	SEM	W	df	*p* Value	Exp (B)	95% CI for Exp (B)	
								Lower	Upper
**Model A** **(without gaming/gambling scores)**	STAI Score	0.029	0.006	22.86	1	0.001	1.03	1.017	1.042
	UPPS-NU	0.118	0.029	16.69	1	0.001	1.13	1.063	1.191
	UPPS-PU	0.089	0.034	6.82	1	0.009	1.09	1.022	1.168
	UPPS-LP	0.044	0.026	2.81	1	0.094	1.05	0.993	1.100
	UPPS-SS	0.074	0.027	7.78	1	0.005	1.08	1.022	1.134
	Hours of use	0.114	0.020	32.65	1	0.001	1.12	1.078	1.166
	AUDIT-C	0.061	0.029	4.53	1	0.033	1.06	1.005	1.125
**Model B** **(including gaming/gambling)**	BDI Score	−0.019	0.010	3.93	1	0.047	0.98	0.963	1.000
	STAI Score	0.032	0.014	5.73	1	0.017	1.03	1.006	1.061
	UPPS-PU	0.151	0.049	9.54	1	0.002	1.16	1.057	1.281
	Hours of Use	0.088	0.034	6.65	1	0.010	1.09	1.021	3.169
	IGDS9-SF Score	0.097	0.016	38.10	1	0.001	1.10	1.068	1.136

## Data Availability

Data availability is granted upon request.
